# Effect of *Ganoderma lucidum* hydroalcoholic extract on insulin release in rat-isolated pancreatic islets 

**Published:** 2012

**Authors:** Reza Shafiee-Nick, Seyyed Mohammad Reza Parizadeh, Nona Zokaei, Ahmad Ghorbani

**Affiliations:** 1 *Pharmacological Research Center of Medicinal Plants, School of Medicine, Mashhad University of Medical Sciences, Mashhad, I. R. Iran*; 2 *Department of Pharmacology, School of Medicine, Mashhad University of Medical Sciences, Mashhad, I. R. Iran*; 3 *Biochemistry and Nutrition Research Center, School of Medicine, Mashhad University of Medical Sciences, Mashhad, I. R. Iran*; 4 *Department of Biochemistry, School of Medicine, Mashhad University of Medical Sciences, Mashhad I. R. Iran*

## Abstract

**Objective:** *Ganoderma Lucidum* (*G.* *Lucidum*) has been suggested to increase serum insulin level. This study was undertaken to investigate its direct effect on the islets of Langerhans.

**Material and Methods:** Male albino Wistar rats were anesthetized and the islets were isolated after digestion of the pancreas with collagenase. The islets were incubated for 60 min in Krebs bicarbonate buffer containing 3 or 10 mM glucose in the presence of hydroalcoholic extract of *G.* *Lucidum* (1 mg/ml), 3-isobutyl-1-methylxanthine (IBMX, 100 µM) or vehicle.

**Results:** Exposure of islets to the extract increased insulin secretion at basal (3 mM) glucose concentration. Increase of glucose concentration to 10 mM resulted in a significant increase in the rate of insulin secretion. While the IBMX could augment insulin release evoked by 10 mM glucose, the extract failed to modify it.

**Conclusion:** Our results demonstrate that *G.* *lucidum* acts directly on the Langerhans islets to increase basal insulin release.

## Introduction

*Ganoderma Lucidum* (*G.* *Lucidum*), a popular medicinal mushroom, is a member of Polyporaceae family that naturally grows on logs of broad-leaf trees (Lin, 2001 ▶). Scientific investigations have confirmed a wide range of biological effects, such as anticancer, immunomodulatory, and antioxidant activities for this mushroom (Lei and Lin, 1992 ▶; Lin, 2001 ▶; Sliva, 2004 ▶; Yuen and Gohel, 2005 ▶). The hypoglycemic effect of *G. Lucidum* was also shown in alloxan- and streptozotocine-induced diabetic rodents (He et al., 2006 ▶; Jia et al., 2009 ▶; Seto et al., 2009 ▶; Xue et al., 2010 ▶; Zhang et al., 2003 ▶).

The hypoglycemic effect of medicinal plants can be achieved through improving and/or mimicking insulin action and/or by enhancing insulin secretion (Gray and Flatt, 1999 ▶). Previous studies have shown the insulinomimetic action of *G. Lucidum* (Hikino et al., 1989 ▶). In parallel, it has been reported that *G. Lucidum* has the ability to increase insulin serum level (Jia et al., 2009 ▶; Zhang et al., 2003 ▶).

Glucose is the most important physiological factor regulating the rate of insulin secretion from pancreatic β-cells. While a basal level of insulin secretion is observed at a sub-threshold level of 3 mM glucose, its secretion is stimulated by a glucose level of >6 mM (Fujimoto et al., 2000 ▶; Shafiee-Nick et al., 1995 ▶). In β-cells, glucose increases intracellular ATP-to-ADP ratio which finally leads to membrane depolarization and Ca^2+^ influx. The rise in intracellular Ca^2+^ concentration triggers insulin secretion (Yamazaki et al., 2010 ▶). Furthermore, glucose increases intracellular cAMP level in the pancreatic islets which may serve to amplify its own primary effect in stimulating insulin secretion. Agents that increase cAMP level, by activating adenylyl cyclase or by inhibiting cAMP phosphodiesterase (PDE), augment glucose-induced insulin release (Seino et al., 2009 ▶). Recently, it has been reported that *G. Lucidum* stimulates insulin secretion (at 5.6 mM glucose) through an increase in intracellular Ca^2+^ concentration, suggesting that further studies are warranted on the insulin secretion mechanism of this plant (Zhang and Lin, 2004 ▶).

The present study was planned to evaluate direct effect of *G. Lucidum* hydroalcoholic extract on insulin release from Langerhans islets in basal and glucose-stimulated condition. Moreover, the possible action of the extract was compared with the effect of 3-isobutyl-1-methylxanthine (IBMX), a non-selective PDE inhibitor.

## Materials and Methods

**Drugs and chemicals**

Bovine serum albumin (BSA) fraction V, collagenase (type IV), dimethyl sulfoxide (DMSO), and IBMX were purchased from Sigma (USA); thiopental sodium was obtained from Sandoz (Austria); Radioimmunoassay kit for insulin test was obtained from Kavoshyar Co. (Iran). All the other chemicals were used of the highest grade available commercially.

**Preparation of** ***G. Lucidum*** **extract**

The *G. Lucidum* were collected from Roodbar jungles in north part of Iran and scientifically approved by the botanists of Ferdowsi University of Mashhad (herbarium no: AZ-B-23). The aerial materials were cleaned, dried at room temperature and grounded to fine powder with a blender. The powder (25 g) was dissolved in ethanol (50% v/v) for 48 h and the extract was then filtered by Buchner funnel under vacuum and dried on a water bath. 

**Isolation of pancreatic islets**

Male albino Wistar rats weighting 250-350 g purchased from the Laboratory Animals House, Mashhad University of Medical Sciences, Iran. Animals were fed standard chow and allowed free access to food and water. They were maintained in a temperature-controlled environment with 12 h light and dark cycles until the experiment. The animals (n=10) were anaesthetized with thiopental (80 mg/kg, i.p.) and the each pancreas was removed following distension with 20 ml cold Krebs bicarbonate buffer (isolation Krebs) (MgSO4 0.9 mM, KH2PO4 1.2 mM, KCl 4.7 mM, NaCl 94 mM, NaHCO_3_ 25 mM, CaCl_2_ 2.5 mM, and glucose 5.6 mM) solution injected via the common bile duct. The tissue was chopped and digested at 37 °C with collagenase (Shafiee-Nick et al., 2011 ▶). Islets were handpicked with a fine, siliconized Pasteur pipette and transferred to Krebs bicarbonate buffer, containing glucose 3 mM, fumarate 5 mM, glutamate 5 mM, pyruvate 5 mM, and BSA 3 mg/ml (incubation Krebs). 

**Incubation of isolated islets**

The islets were placed in vials containing 1 ml incubation Krebs and pre-incubated in a shaking incubator (60 oscillations/min) with continuous gassing (95% 0_2_, 5% CO_2_, pH 7.4, 37 °C). The medium was removed and replaced by 1 ml fresh incubation Krebs solution containing the extract, IBMX (100 µM) or vehicle in the presence of 3 or 10 mM glucose. Working solution of the extract was prepared by the incubation Krebs to make a final concentration of 1 mg/ml. IBMX was prepared as stock solutions in DMSO and diluted in Krebs buffer. The appropriate quantity of DMSO was used as vehicle. The islets were incubated for 60 min at 37 °C. Then, a sample of the medium was removed and frozen for subsequent determination of insulin. Four to five batches of islets were used for each treatment. The experiment was performed using 4-5 different isolates (two pancreata from two rats being used for each isolate). 

**Insulin assay**

The Insulin level was measured using a commercial human radioimmunoassay kit containing a polyclonal antibody with 100% cross-reactivity for rat insulin.

**Statistical analysis**

Statistical significance was determined by one-way ANOVA. Any p-value below 0.05 was considered significant. Results obtained are expressed as mean±SEM.

## Results

**Effect of** ***G. Lucidum*** **on basal insulin secretion**

**Figure 1:**
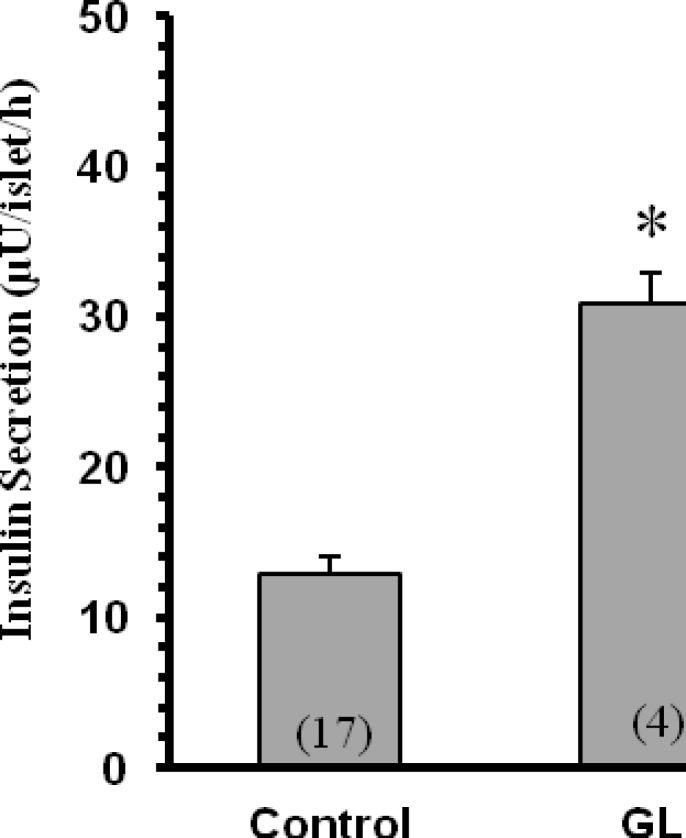
Effect of *Ganoderma lucidum* on insulin secretion by rat-isolated pancreatic islets in the presence of 3 mM glucose. The islets were incubated for 60 min in the Krebs solution containing 3 mM glucose in the absence (control) or presence of 1 mg/ml *Ganoderma lucidum* (GL) extract. Each value is mean±SEM of the numbers shown in parentheses. The quoted *n* values refer to 4-5 batches of islets from 4-5 different isolates (with two pancreata being used for each isolate). *p<0.001 versus control

As shown in Figure 1, in the buffer containing 3 mM glucose, the rate of basal insulin secretion was 12.9±1.2 µU/islet/h. In the presence of 3 mM glucose, the extract (1 mg/ml) significantly increased insulin secretion (24.9±2.8 µU/islet/h, p<0.05).

**Effect of** ***G. Lucidum*** **on glucose stimulated insulin secretion**

**Figure 2:**
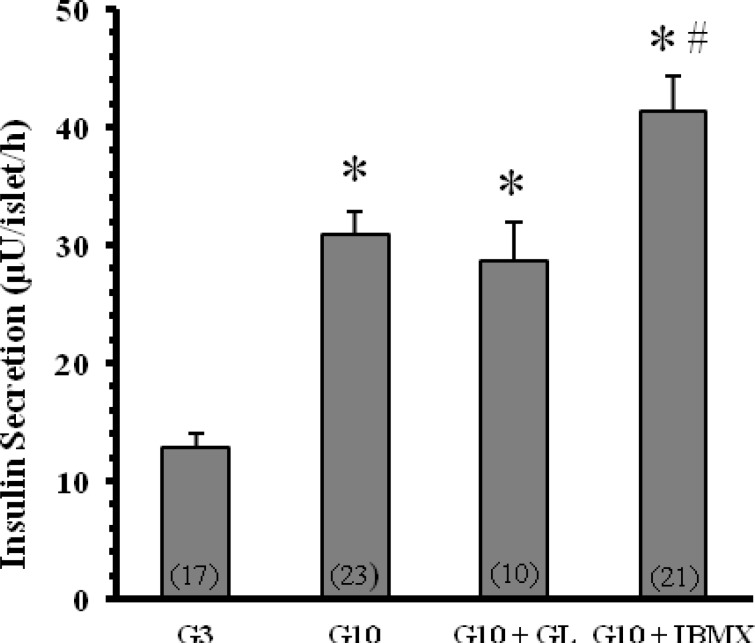
Effect of *Ganoderma lucidum* and IBMX on insulin secretion by rat-isolated pancreatic islets in the presence of 10 mM glucose. The islets were incubated for 60 min in the Krebs solution containing 3 mM (G3) and 10 mM glucose (G10) in the absence or presence of 1 mg/ml *Ganoderma lucidum* (GL) extract or 100 µM 3-isobutyl-1-methylxanthine (IBMX). Each value is mean±SEM of the numbers shown in parentheses. The quoted *n* values refer to 4-5 batches of islets from 4-5 different isolates (with two pancreata being used for each isolate). *p<0.001 versus G3; ^#^p< 0.05 versus G10.

Increase of glucose concentration from 3 to 10 mM resulted in a significant increase (30.9±2 µU/islet/h, p<0.001) in the rate of insulin secretion. The insulin release evoked by 10 mM glucose was augmented (41.4±3 µU/islet/h, p<0.05) by 100 µM IBMX. However, the *G. Lucidum* extract failed to modify the insulin-releasing effect of 10 mM glucose (28.7±3.2 µU/islet/h) (Figure 2).

## Discussion

It has been shown that *G. Lucidum* has the ability to increase insulin serum level in rodents (Jia et al., 2009 ▶; Zhang et al., 2003 ▶; Hikino et al., 1989 ▶). Therefore, in the current research, authors found it of interest to study direct effects of this plant on insulin secretion using rat-isolated pancreatic islets technique. First, the responsiveness of the isolated islets to physiological and pharmacological agents was checked. Our observation that insulin release was stimulated by increase of glucose in medium confirms that the islets were physiologically responsive. Furthermore, the IBMX could further augment insulin release evoked by glucose, indicating that the islets were also sensitive to pharmacological factors. Using the isolated islets, our data demonstrate that the insulinotropic action of G. *lucidum* occurs only in basal (3 mM) glucose concentration, similar to fasting condition. The finding is consistent with earlier research showing that in fasted mice, the hypoglycemic effects of *G.* *lucidum* polysaccharides are related to insulin release by pancreatic β-cells (Zhang and Lin, 2004 ▶). Therefore, it seems that the extract has positive effect on glucose-sensing process in β-cells. As this process become impaired in type-2 diabetes (Thorens, 2008 ▶), the extract may have beneficial effects on the patients. Moreover, we showed that the extract failed to augment 10 mM glucose induced-insulin-releasing. The finding suggests that the glucose lowering effect of the *G. Lucidum* in postprandial condition may result from extra-pancreatic effects. Consistent with this hypothesis, Hikino et al. reported that Ganoderan B, a glycan of *G.* *lucidum*, modulates hepatic enzyme activities (Hikino et al., 1989 ▶). 

The molecular mechanism responsible for *G.* *lucidum*-induced insulin secretion is not well understood. In this study, we tried to gain more insight into the likely mechanisms implicated for insulinotropic action of *G.* *lucidum*. Although the best known mechanism underlying insulin secretion is increase of ATP-to-ADP ratio, the secretion can be stimulated through other ways such as cAMP-dependent pathway (Seino et al., 2009 ▶; Yamazaki et al., 2010 ▶). For example, some agents, such as IBMX, that elevate intracellular cAMP, by inhibiting cAMP phosphodiesterase, markedly augment glucose-induced insulin release (Shafiee-Nick at al., 1995 ▶). Consistent with this fact, we observed that a combination of IBMX and glucose (10 mM) produced a synergisticm effect in augmentation of insulin release; confirming that they modulate insulin secretion through different pathways. Our finding that the extract did not further augment the insulinotropic effect of 10 mM glucose, suggests that the mechanism by which *G.* *lucidum* stimulates insulin secretion is not different from that of 10 mM glucose. The hypothesis is in accordance with the results of Zhang and Lin who demonestrated that *G. Lucidum* stimulates insulin secretion (at 5.6 mM glucose) through an increase in intracellular Ca^2+^ concentration. Beside, in prolonged consumption, the plant can also increase insulin release by its scavenging ability to protect the pancreatic islets against free radicals damages (Jia et al., 2009 ▶; Zhang et al., 2003 ▶).

In summary, our data demonstrate that G. *lucidum* acts directly on the Langerhans islets to increase basal insulin release. This finding presents a possible mechanism for the hypoglycemic effect of G. *lucidum* reported by previous studies.

## References

[B1] Fujimoto S, Tsuura Y, Ishida H, Tsuji K, Mukai E, Kajikawa M, Hamamoto Y, Takeda T, Yamada Y, Seino Y (2000). Augmentation of basal insulin release from rat islets by preexposure to a high concentration of glucose. Am J Physiol Endocrinol Metab.

[B2] Gray AM, Flatt PR (1999). Insulin-releasing and insulin-like activity of the traditional anti-diabetic plant Coriandrum sativum (coriander). Br J Nutr.

[B3] He CY, Li WD, Guo SX, Lin SQ, Lin ZB (2006). Effect of polysaccharides from Ganoderma lucidum on streptozotocin-induced diabetic nephropathy in mice. J Asian Nat Prod Re.

[B4] Hikino H, Ishiyama M, Suzuki Y, Konno C (1989). Mechanisms of hypoglycemic activity of Ganoderan B: a glycan of Ganoderma lucidum fruit bodies. Planta Med.

[B5] Jia J, Zhang Xi, Hu YS, Wu Y, Wang QZ, Li NN, Guo QC, Dong XC (2009). Evaluation of in vivo antioxidant activities of Ganoderma lucidum polysaccharides in STZ-diabetic rats. Food Chem.

[B6] Lei LS, Lin ZB (1992). Effects of Ganoderma polysaccharides on T cell subpopulations and production of interleukin 2 in mice lymphocyte response. Yao Xue Xue Bao.

[B7] Lin ZB (2001). Modern research of Ganoderma lucidum.

[B8] Seino S, Takahashi H, Fujimoto W, Shibasaki T (2009). Roles of cAMP signaling in insulin granule exocytosis. Diabetes Obes Metab.

[B9] Seto SW, Lam TY, Tam HL, Au ALS, Chan SW, Wu JH, Yu PHF, Leung GPH, Ngai SM, Yeung JHK, Leung PS, Lee SMY, Kwan YW (2009). Novel hypoglycemic effects of Ganoderma lucidum water-extract in obese/diabetic (+db/+db) mice. Phytomed.

[B10] Shafiee-Nick R, Pyne NJ, Furman BL (1995). Effects of type-selective phosphodiesterase inhibitors on glucose-induced insulin secretion and islet phosphodiesterase activity. Br J Pharmacol.

[B11] Shafiee-Nick R, Parizadeh SMR, Zokaei N, Ghorbani A (2011). Effect of hydro-alcoholic extract of Vaccinium arctostaphylos on insulin release from rat-isolated langerhans islets. Koomesh.

[B12] Sliva D (2004). Cellular and physiological effects of Ganoderma lucidum (rishi). Mini Rev Med Chem.

[B13] Thorens B (2008). Glucose sensing and the pathogenesis of obesity and type 2 diabetes. Int J Obes.

[B14] Xue H, Qiao J, Meng G, Wu F, Luo J, Chen H, Zheng H, Xu J (2010). Effect of Ganoderma lucidum polysaccharides on hemodynamic and antioxidation in T2DM rats. Zhongguo Zhong Yao Za Zhi.

[B15] Yamazaki H, Zawalich KC, Zawalich WS (2010). Physiologic implications of phosphoinositides and phospholipase C in the regulation of insulin secretion. J Nutr Sci Vitaminol.

[B16] Yuen JWM, Gohel MDI (2005). Anticancer effects of Ganoderma lucidum: a review of scientific evidence. Nutr Cancer.

[B17] Zhang HN, He JH, Yuan L, Lin ZB (2003). In vitro and in vivo protective effect of Ganoderma lucidum polysaccharides on alloxan-induced pancreatic islets damage. Life Sci.

[B18] Zhang HN, Lin ZB (2004). Hypoglycemic effect of Ganoderma lucidum polysaccharides. Acta Pharmacol Sin.

